# High ceftazidime-avibactam resistance rate in carbapenem-resistant gram-negative organisms in Pakistan’s pediatric population

**DOI:** 10.12669/pjms.41.3.9978

**Published:** 2025-03

**Authors:** Naima Mehdi, Nadia Majeed, Farwa Ali, Iqra Aroob

**Affiliations:** 1 Naima Mehdi, M.Phil. Assistant Professor, Department of Microbiology, University of Child Health Sciences, The Children’s Hospital, Lahore, Pakistan; 2 Nadia Majeed, FCPS. Assistant Professor, Department of Infectious Diseases, University of Child Health Sciences, The Children’s Hospital, Lahore, Pakistan; 3 Farwa Ali, BS. Research Assistant, Department of Microbiology, University of Child Health Sciences, The Children’s Hospital, Lahore, Pakistan; 4 Iqra Aroob, Ph.D. Assistant Professor, School of Allied Health Sciences, University of Child Health Sciences, The Children’s Hospital, Lahore, Pakistan

**Keywords:** Antimicrobial resistance, Carbapenemases, Ceftazidime-Avibactam, ESBLs, Gram-Negative Organisms

## Abstract

**Objective:**

This study investigates resistance to a newly available drug Ceftazidime–Avibactam (CAZ-AVI) in carbapenem-resistant gram-negative organisms (GNOs) in the Pakistan’s pediatric population.

**Methods:**

This was a prospective study carried out in the Department of Microbiology at The Children’s Hospital, Lahore from May 2023 to July 2023. A sum of 7491 specimens of blood, cerebrospinal fluid (CSF), urine, pus, nasal swabs, central venous catheter (CVP) tips and tracheal secretion were analyzed for presence of carbapenem resistant gram-negative organisms which were then further screened for CAZ-AVI resistance. Analytical profile index and Kirby-Bauer disk diffusion methods were used for the identification of organisms.

**Results:**

A total of 217(n) carbapenem-resistant bacterial species, including 165(n) *Klebsiella* sp., 32(n) *Pseudomonas sp*., and 20(n) *Escherichia coli* (*E. coli*) strains, were tested for sensitivity against CAZ–AVI. Approximately, 70.5% (153 out of 217) of carbapenem-resistant bacteria exhibited resistance to CAZ-AVI. Among the resistant bacterial species, 80% (122/153) were *Klebsiella* sp., 14% (21/153) were *Pseudomonas* sp., and 6% (10/153) were *E. coli*. These findings suggest pre-existing resistance mechanisms may be responsible for exhibiting resistance to CAZ-AVI in these organisms.

**Conclusion:**

Taking into account the results of this study, which depicted high resistance rates to CAZ-AVI among carbapenem resistant GNOs, and the high cost of this drug, it is suggested that a cautious selection for its use as monotherapy in sepsis and other infections should be made. It should be made mandatory to check the resistance to CAZ-AVI before its empiric use. The findings of the study also emphasize the challenges of combating new drug resistance and further research to adapt treatment strategies to the evolving antimicrobial resistance in the region.

## INTRODUCTION

Severe clinical infections caused by multidrug-resistant (MDR) bacteria pose a significant threat to pediatric patients in Pakistan.^1^ Treatment options for infections caused by β-lactamase producing gram-negative organisms (GNOs), particularly those involving carbapenem-resistance, are limited.^2^ However, in February 2015, the approval by the US Food and Drug Administration (FDA) of a novel cephalosporin/β-lactamase inhibitor combination, Ceftazidime–Avibactam (CAZ-AVI), significantly mitigated concerns associated with conventional treatment approaches for multidrug-resistant (MDR) infections caused by such organisms.^3^,^4^

Avibactam is a member of a class of β-lactamase inhibitors called diazabicyclooctanes (DBOs).^5^ It has the capacity to rapidly acylate a wide range of β-lactamases. CAZ–AVI thus exhibits activity against various clinically important β-lactam-resistant organisms including streptococcal species, gram negative bacilli including, *Escherichia coli* (*E. coli*), *Klebsiella pneumonia*, *Proteus mirabilis*, *Providencia sp*. *Salmonella sp*. *Serratia marcescens*, *Capnocytophaga sp*., *Hemophilus influenzae*, *Kingella sp*., *Vibrio sp*. etc.^6^ However, bacterial resistance poses a potential risk with antibacterial usage. In the short period since the introduction of CAZ–AVI in clinical use, reports of bacterial pathogens developing resistance have surfaced worldwide.^7^-^10^ Therefore, there is an urgent need to address the emergence of CAZ–AVI resistance during treatment.

In Pakistan, CAZ-AVI was not available as a treatment option until 2022. Once it became accessible for use in our population, this study was conducted to assess the resistance pattern of CAZ-AVI on carbapenem-resistant GNOs isolated from various samples of pediatric patients at The Children’s Hospital, Lahore.

## METHODS

This was a prospective study carried out in the Department of Microbiology at The Children’s Hospital, Lahore. Inclusion criteria included all of the GNOs identified during the study duration of three months from May 2023 to July 2023. A sum of 7491 specimens of blood, cerebrospinal fluid (CSF), urine, pus, nasal swabs, central venous catheter (CVP) tips and tracheal secretion were analyzed for presence of carbapenem resistant GNOs which were then further screened for CAZ-AVI resistance.

### Ethical approval:

The research has been performed following the Declaration of Helsinki. The ethical approval was granted by the Institutional Review Board (IRB), University of Child Health Sciences, The Children’s Hospital, Lahore, at April 26, 2023 under IRB approval Letter Number: 651/UCHS-CH.

All types of medias and biochemical reagents were obtained from either Oxoid ltd (ThermoFisher Scientific), Condalab, or Neogen®. Antimicrobial sensitivity discs were obtained from Bioanalyse®. API 20E were manufactured by bioMerieux (France). MacConkey agar (pH 7.1±0.2 at 25 °C) and Muller-Hinton agar (pH 7.4±0.2 at 25 °C) were used for identification of bacterial species and sensitivity tests on antibiotics, respectively. GNOs were identified based on their morphology, Gram staining and standard biochemical testing for *Klebsiella* sp., *Pseudomonas* sp. and *E.coli*. The Analytical Profile Index API 20E (Biomerieux, France) were utilized for the identification of lactose-fermenting and non-lactose-fermenting species. A suspension of the test organism was prepared by mixing a well-isolated colony in saline water. Using a sterile syringe, the suspension was inoculated into the tubes of the strip. The tubes were covered and incubated at 37°C for 18-24 hours. Identification was performed using a database with the APIWEB™ identification software by entering the numerical code.

The antimicrobial susceptibility of the isolates was assessed using the Kirby-Bauer disk diffusion method^11^,^12^ for both carbapenem and CAZ-AVI. Four to five isolated colonies exhibiting similar morphology were taken and suspended in 4-5 ml of sterile saline solution, adjusted to a turbidity equivalent to the 0.5 McFarland standard. The suspension was swabbed onto the entire Mueller Hinton Agar (MHA) plates three times, turning the plate 60° between streaking to ensure even inoculation. Using a multi-dispenser, commercially available antibiotic discs were added within 15 minutes, and the plates were incubated at 37°C for 16-20 hours. GNOs were tested against carbapenems and CAZ-AVI. The interpretation of a clear inhibition zone was conducted following CLSI guidelines in 2023. For carbapenems a zone size of <=19 was considered resistant while a zone size of >=23 was considered susceptible. Similarly for CAZ-AVI a zone size of <=20 was considered resistant while a zone size of >=21 was considered susceptible.

## RESULTS

### Identification of bacterial isolates:

Of the 7491 samples processed for identification of pathogenic strains, 1970 were identified as GNOs (appeared pink during gram staining) and 416 were identified as gram-positive organisms (GNPs). After the initial identification using morphological analysis, biochemical testing and API profiles, cultures that tested positive for *Klebsiella* sp., *Pseudomonas* sp. and *E.coli* were further analyzed to assess resistance to carbapenem and CAZ-AVI.

### Drug Sensitivity Testing:

**Fig.1:**
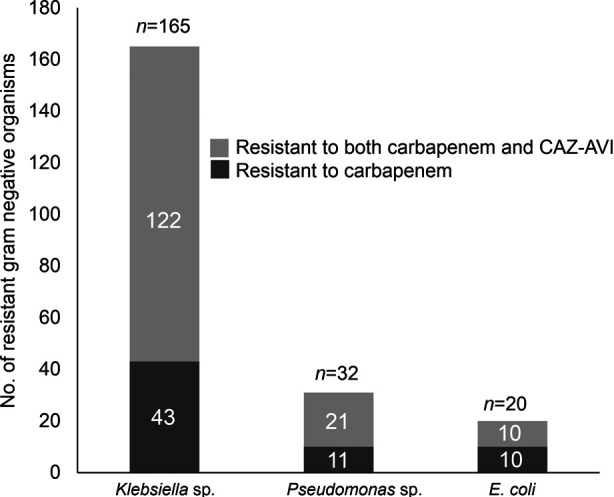
Bar graph showing number of gram-negative organisms resistant to carbapenem or both carbapenem and CAZ-AVI.

Among the selected 1970 GNOs, a total of 217 were identified as carbapenem-resistant. Within this group, 165 were classified as *Klebsiella* sp., 32 as *Pseudomonas* sp. and 20 as *E. coli* (Fig.1). Of these 217 carbapenem-resistant isolates, 153 exhibited resistances to CAZ-AVI. Specifically, within the bacteria resistant to both carbapenem and CAZ-AVI (n=153), 80% (122/153) were identified as *Klebsiella* sp., 14% (21/153) as *Pseudomonas* sp., and 6% (10/153) as *E. coli* (Fig. 1). This resulted in an overall high resistance rate of 70.5% (153/217) of carbapenem-resistant GNOs against this novel combination of cephalosporin and β-lactamase inhibitor.

## DISCUSSION

Carbapenems continue to be used for the treatment of infections caused by GNOs as suggested by both, EUCAST (European Committee on Antimicrobial Susceptibility Testing)(http//www.eucast.org)^13^ and CLSI (Clinical and Laboratory Standards Institute) guidelines.^14^ However, carbapenem resistance is becoming an increasingly severe threat to public health worldwide. The rise of carbapenem resistance poses a significant challenge to the global public health network because clinical infections caused by carbapenem-resistant pathogens are often linked to high rates of morbidity and mortality.

CAZ-AVI is a novel cephalosporin/β-lactamase inhibitor combination offering an important advance in the treatment of carbapenem-resistant pathogenic infections. In February 2015, the FDA gave approval of CAZ–AVI, which alleviated many of the concerns regarding traditional treatment options for multi-drug resistant gram negative bacterial infections.^2^-^4^

In this study, we have tested carbapenem-resistant GNOs isolated from blood, urine or pus samples of pediatric patients to the newly available drug, CAZ-AVI. We tested a total of 217 carbapenem-resistant GNOs, which included 165 *Klebsiella* sp., 32 *Pseudomonas* sp., and 20 *E. coli* strains. Among these 217 isolates, 153 were found to be resistant to CAZ-AVI, comprising 122 *Klebsiella* sp., 21 *Pseudomonas* sp. and 10 *E. coli*. This resulted in a very high resistance rate of 70.5% for this new drug.

Reports on bacterial resistance to CAZ-AVI have been emerging shortly after the introduction of this drug in clinical use. The first case of a *Klebsiella pneumoniae* isolate demonstrating resistance to CAZ-AVI was documented in a patient at UCLA Medical Center, Los Angeles, CA, who had not previously received CAZ-AVI therapy in the same year when the use of drug was approved.^9^,^15^ Followed by this report, a report showing resistance against CAZ-AVI in *Klebsiella* was published from China.^16^ Further reports from the Europe also showed strains resistant to CAZ-AVI.^17^ In Pakistan, resistance to CAZ-AVI has recently been reported from Karachi^18^ and Lahore^19^ regions from the clinical settings having patient influx from all age groups. Similar to the high resistance to CAZ-AVI shown by carbapenem resistant GNOs in our study, the other two studies^18^,^19^ have also indicated a high resistance to CAZ-AVI. On contrary to very high resistance to CAZ-AVI being reported from Pakistan, the studies on CAZ-AVI resistance from China have reported comparatively lower resistance rates of 5.4% and 13.5% for Enterobacterales and *P. aeruginosa* respectively.^20^

CAZ-AVI is a recently marketed antibiotic in Pakistan and is among the most expensive antibiotics available. In practice physicians may inadvertently overuse newly launched antibiotics without knowing their antimicrobial spectrum and resistance pattern. NDM is common β-lactamase in our region and CAZ-AVI is ineffective against this. Our study is conducted in pediatric population on bacterial strains which were resistant to carbapenems where there can be potential empiric use of CAZ-AVI. We have documented the resistant pattern in these strains to highlight the need for cautious empirical use of CAZ-AVI. Until sensitivity patterns are confirmed, this drug should not be used as monotherapy. To the best of our knowledge, resistance to CAZ-AVI among the pediatric population has not been previously studied or documented in Pakistan, although a similar study with comparable findings has been conducted in the adult population.^18^

Previous studies have identified that CAZ-AVI resistance is associated with different β-lactamases such as ESBL types, OXA-48, NDM and KPC.^8^ In our region, Pakistan, bla NDM-1 and bla KPC-2 in clinical isolates of *Klebsiella pneumoniae* is already extensively reported^21^-^24^, which can be the possible explanation of already existing resistance against CAZ-AVI. The findings in this study will provide an opportunity for PCR based identification of specific β-lactamases responsible for conferring CAZ-AVI resistance in our population, especially pediatric population.

### Limitations:

Although the study provides very significant findings regarding resistance to CAZ-AVI in our patents even before its use, but we were unable to identify the genes which were conferring Gram-negative organisms resistant to CAZ-AVI.

## CONCLUSION

The study finds a high resistance rate to CAZ-AVI in our region. CAZ-AVI is already being over-prescribed by physicians and therefore the findings of our study provide guidance to healthcare providers regarding its suitability as monotherapy for sepsis and other severe infections caused by GNOs.

### Recommendations:

Careful selection of CAZ-AVI should be made for treatment of infections caused by carbapenem-resistant GNOs. It is also suggested to check the resistance to CAZ-AVI before its empiric use.

### Authors’ Contributions:

**Naima Mehdi:** Conceptualization, Study Design, Supervision, Reviewing Manuscript.

**Nadia Majeed:** Conceptualization, Study Design, Reviewing Manuscript.

**Farwa Ali:** Experimentation, Data Collection. Critical Review.

**Iqra Aroob:** Data Curation, Data Analysis, Writing and revising the manuscript. All authors have read the final version to be published and are accountable for integrity of the study.
